# The growth and standing of Australian psychology research: a snapshot

**DOI:** 10.1080/00049530.2025.2485473

**Published:** 2025-04-02

**Authors:** Nick Haslam

**Affiliations:** Melbourne School of Psychological Sciences, University of Melbourne, Melbourne, Australia

**Keywords:** Australia, psychology, research funding, research metrics

## Abstract

**Objective:**

Australian psychology research has become globally prominent in recent years. The present study aimed to quantify its growth and describe its current standing nationally and internationally.

**Method:**

Four databases were consulted to quantify historical trends in psychology publications by Australian-affiliated authors and to characterise the current standing of the field in terms of publications, leading researchers and research funding.

**Results:**

Australian-affiliated researchers have produced a steeply rising proportion of psychology articles since 1970. They now account for 5.8% of global productivity, a rate that exceeds most other fields of research in Australia and most leading nations when adjusted for population. Quality, assessed by top-quartile journal publications, is also high by international standards. Australia’s share of leading psychology researchers is more modest. Approximately 5.7% of grant and fellowship funding from the Australian Research Council goes to psychology researchers, with stronger outcomes for fellowships than for industry-partnered grants.

**Conclusions:**

Psychology research in Australia is a success story. In the past half-century, the country has emerged as a major producer of psychological knowledge with a profile that stands out internationally and, in comparison with other fields, nationally. Whether its success is sufficiently recognised or rewarded is open to debate.

## Key Points

**What is already known about this topic:**

(1) Psychological science in Australia has a long and complex history.
(2) Psychology research has grown with the expansion of the Australian university sector.
(3) Australian psychology research has become increasingly prominent internationally.

**What this topic adds:**

(1) Australian-affiliated psychology research productivity has risen steeply since the 1970s and now accounts for 5.8% of all articles.
(2) On a population-adjusted basis, Australian psychology research productivity exceeds most traditional research powerhouses.
(3) Psychology is one of Australia’s most prominent fields of research based on its share of global publications.

## Introduction

The rapid expansion of psychology’s footprint in Australian universities has been accompanied by a rise in the country’s contributions to psychological science. Many Australian psychology researchers are internationally prominent, hold editorial and leadership roles in major journals and organisations, and compete successfully for research funding with their peers in more long-established scientific fields. Psychology reached maturity relatively late in Australia where, as a notionally social science, it was something of a “poor relation” (Macintyre, 2010) to the natural and technological sciences. However, its rise has been steep.

The history and contemporary state of Australian psychology research have been explored on many occasions and in many ways. Periodic overviews have examined the discipline as a whole (e.g., Taft, 1988; Turtle, 1985) or specific subdisciplines (e.g., Feather, 2005). Some investigations have taken a critical perspective, pointing to the field’s weaknesses and limitations. Breen and Darlaston-Jones (2010) chastised it for its excessive commitment to positivism, and Sheehan (1996), citing a review that year of the discipline by the Australian Research Council (ARC), challenged the monocultural outlook, neglect of ethical and epistemological issues, and limited social relevance of Australian research.

Explorations such as these have been qualitative in nature, identifying topics, themes, and key events, concepts and people, with the exception of a few studies of local publication and citation norms (e.g., Mazzucchelli et al., 2019). Some writers have pointed to quantitative trends in Australian psychology research without measuring them. Turtle (1985), for example, observed that the early 1980s were a time of limited growth after the “burgeoning” of the 1960s and 1970s. In view of the absence of data-driven studies of the field, a quantitative overview of Australian psychology research is overdue.

To that end, the present study offers a snapshot of the development and status of psychology research in Australia, with a focus on publications, researchers, and research funding. First, it charts the growth of the country’s publication output over the past half-century, anticipating steep increases in absolute terms and as a share of global production. Second, it assesses the extent and quality of the country’s current publication footprint and its share of internationally leading researchers. Third, it compares these quantities to other research fields, asking whether psychology is a field of relative strength in the national context. Finally, it evaluates the proportion of nationally competitive research funding for basic and applied science that supports psychology research, and whether it differs across major funding schemes.

## Method

We consulted four databases to provide an overview of the development and current standing of Australian psychology research. Web of Science was used to quantify publications in journals in the “Psychology” category and its subdisciplinary categories, restricted to document types “article” or “review article”, from its inception in 1970 through to 2024, sorted by publication year. Journal Citation Reports were consulted for the latest (2023) journal impact factors. The August 2024 data-update of a Scopus-sourced database of standardised citation indicators (Ioannidis, 2024) was used to identify leading psychology researchers with Australian affiliations. The ARC’s website was used to source information on research funding outcomes for 2022–2024, averaging over the three years to level out year-by-year fluctuations. Our focus was restricted to nonmedical psychology research, so outcomes from the National Health and Medical Research Council (NHMRC) and the Medical Research Future Fund (MRFF) were not examined.

## Results

### Historical changes in publication volume

**Figure 1.**
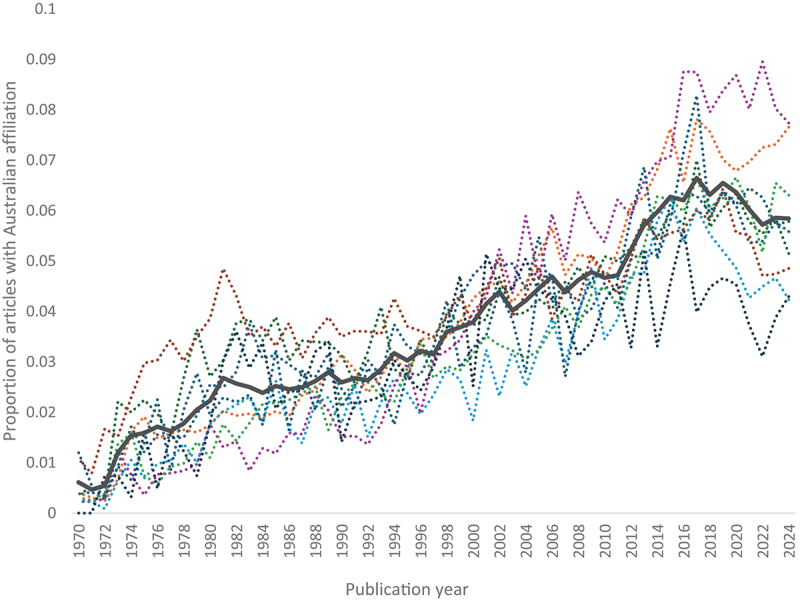
Proportion of articles with an Australian affiliation for psychology as a whole (solid line) and its subfields (dotted lines) (1970–2024).

Figure 1 presents the proportion of all publications in Web of Science-listed “Psychology” journals (solid line) and its subfields (dotted lines) that have a listed Australian author affiliation (publications may have more than one national affiliation). It reveals a steady rise from the early 1970s to a peak of 6.5% of articles in 2019, followed by a slight decline. The rise is relatively uniform across the nine subfields recognised by Web of Science. In 2024, the highest proportions of Australian-affiliated articles were in applied and clinical psychology (both 7.7%), and the lowest in educational (4.2%) and mathematical (4.3%) psychology. Mean productivity differs across subfields in Australia (Haslam et al., 2017), but although these differences affect the weighting of each subfield’s contribution to the “Psychology” proportion they do not bias the within-subfield comparisons on which these proportions are based.

### Current standing

#### Publications

**Figure 2.**
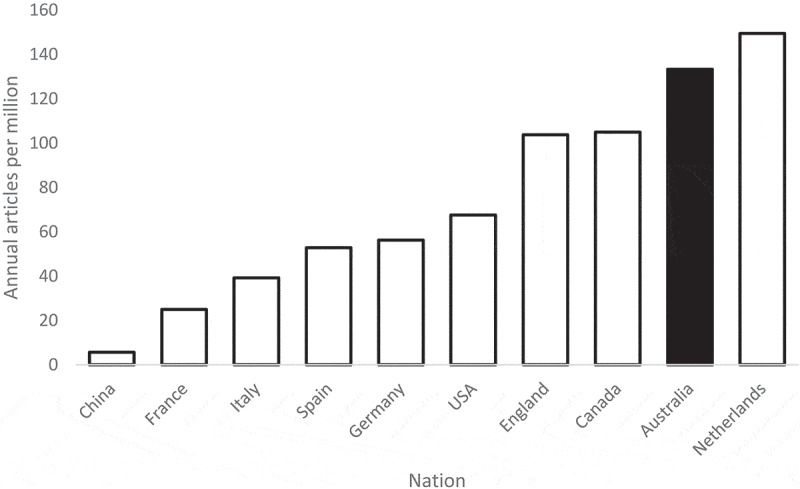
Population-adjusted production of psychology articles for ten most productive nations (2022–2024).

Figure 1 indicates that 5.8% of psychology articles published in the period 2022–2024 had Australian-affiliated authors. Figure 2 presents population-adjusted rates (annual publications per million residents) for the same period among the 10 nations with the highest publication counts according to Web of Science. World Bank population estimates for 2023 were used for all nations except England, for which an estimate (from 2022) was sourced from the UK Office of National Statistics. Australia comes second only to the Netherlands on this metric.

**Figure 3.**
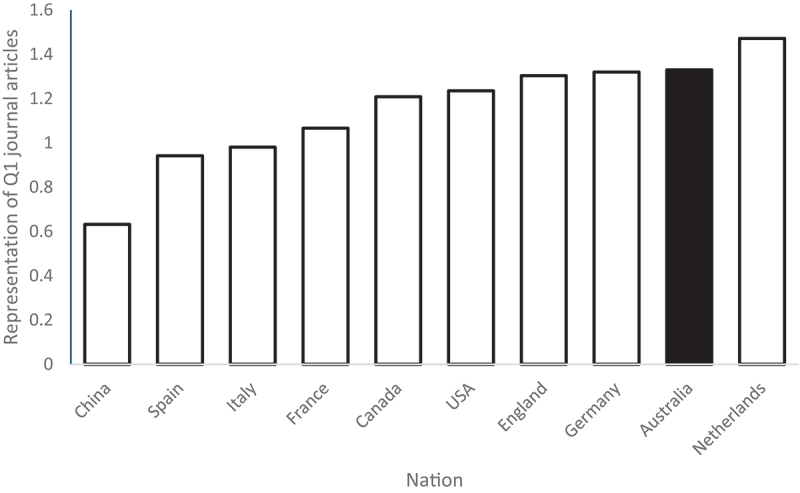
Proportion of articles in Q1 journals relative to proportion of all articles for ten most productive nations (2022–2024).

As a proxy of publication quality, all top-quartile (Q1) “Psychology” journals were identified based on 2023 Journal Citation Reports. Database limitations restricted the analysis to the 200 journals (all quartiles) with the most articles for the period 2022–2024. The proportion of Australian-affiliated articles in these journals (5.93%) was compared to the proportion of the Australian-affiliated articles in the Q1 journal subset (7.89%), indicating that Australian authors were over-represented in Q1 journals by a factor of 1.33. The magnitude of this over-representation is presented in Figure 3. Again, Australia’s over-representation compares favourably to most other leading nations.

**Figure 4.**
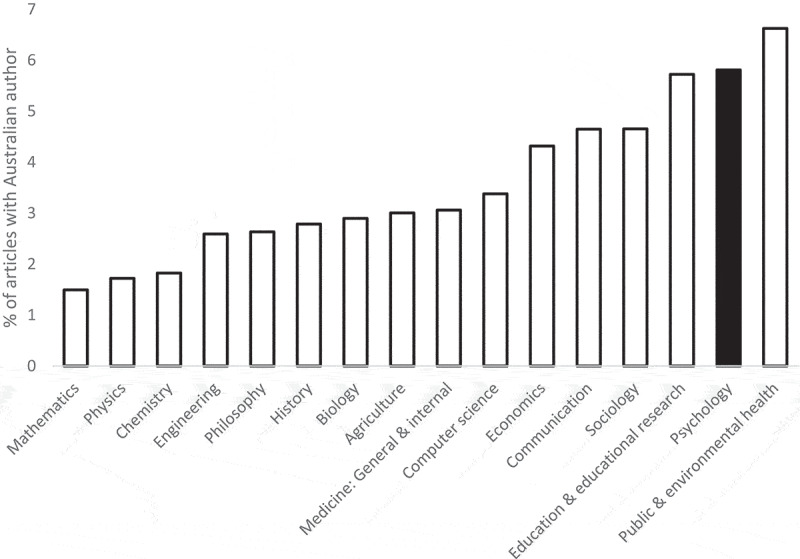
Percentage of articles with an Australian affiliation by field (2022–2024).

Figure 4 compares the proportion of Australian-affiliated articles across selected Web of Science fields rather than nations, again based on 2022–2024 data. The figure shows that Australia accounts for a higher share of global articles in psychology than most fields, indicating that it is a field of relative national strength.

#### Leading researchers

**Figure 5.**
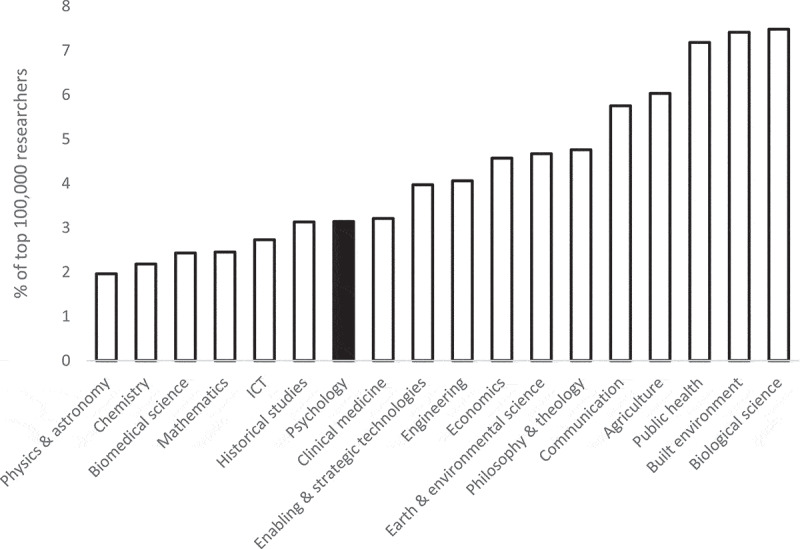
Percentage of top 100,000 researchers with an Australian affiliation by field (2024).

Whether Australia also has relative strength in leading psychology researchers is also pertinent. Ioannidis and colleagues (2019, 2020) have developed an annually updated researcher database which includes a composite citation-based metric that enables relatively equitable comparison across fields. Using Scopus data, it ranks the top 100,000 researchers on this metric. Of the 3,380 Australian-affiliated researchers, 107 (3.17%) have “psychology” listed as their primary field. Figure 5 displays the proportion of top 100,000 Australian-affiliated researchers in all major fields in the database. The psychology proportion (3.14%) exceeds those for physical and mathematical sciences but is lower than many other fields. It is noteworthy that the rank ordering of fields resembles Figure 4 – although their respective Web of Science and Scopus field classifications do not align perfectly – but Australian psychology ranks much lower on the top researcher measure.

#### Funding

**Figure 6.**
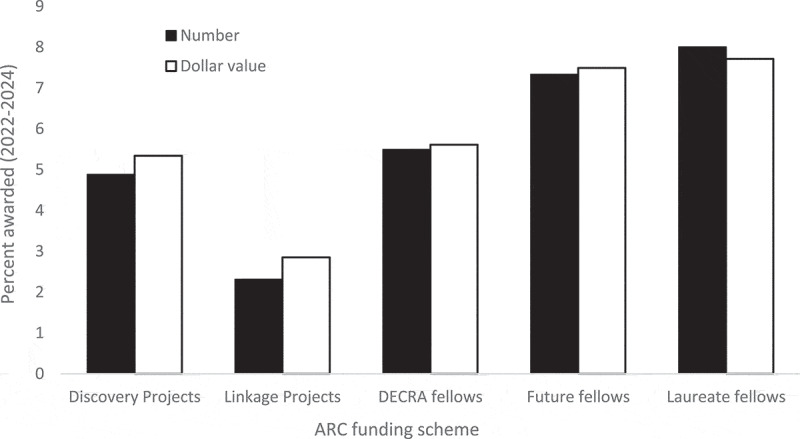
Percentage of Australian Research Council grants and fellowships awarded to psychology (2022–2024).

Finally, to evaluate the proportion of nationally competitive basic and applied science funding that flows to psychology research, we examined published grant and fellowship outcomes from the Australian Research Council (ARC). In the period 2022-2024, 2,774 grants or fellowships worth $1.699 billion dollars were awarded. Of these, 138 (4.98%) valued as $96.71 million (5.69%) went to psychology projects (identified as ANZRC Field of Research division 17 [2022] or 52 [2022-2024]). Figure 6 summarises the proportion of awards by number and value for five major ARC schemes, showing higher proportions for fellowships than grants and especially than industry-engaged Linkage Projects.

## Discussion

Our findings offer a quantitative overview of the growth and contemporary status of psychology research in Australia. That growth has been vigorous, outpacing the enlargement of the field internationally (as indexed by annual publications) almost by a factor of 10. In 1970, 65 articles from a global pool of over 10,000 had an Australian affiliation, compared to more than 3,000 articles out of almost 60,000 in 2024. In parallel with the rapid expansion of the Australian tertiary education sector, psychology researchers and their works have proliferated, and have done so in ways that are relatively even across subfields. The extent of this rise is revealed when Australia’s publication outputs are population-adjusted. By this metric the country outperforms traditional North American and British powerhouses. This result may point to strong pressures to publish or a relatively undifferentiated tertiary education system in which all universities value research. The finding that Australian researchers are over-represented in Q1 journals implies that incentives to pursue quantity are not seriously compromising quality.

Cross-field comparisons further indicate that Australian psychology research achieves a greater share of global publications than most research fields. By implication, psychology is a field in which the country over-achieves, a fact that may not be sufficiently appreciated. Australia’s research footprint in natural sciences like physics and chemistry is much smaller by global standards, for example, but these fields enjoy a sizeable advantage in recognition by prestigious awards (Haslam & Baes, 2023). Producing a relatively small share of global research does not imply that share is unimportant. However, fields in which the country has greater prominence should be acknowledged and supported as areas of competitive advantage.

The proportion of Australian psychology researchers who are international leaders according to whole-of-career citation metrics is much smaller than would be expected based on the proportion of Australian-authored publications. This finding suggests that Australia’s strong publication output is less dependent on the work of senior researchers in psychology than in other fields. It may represent a less unequal distribution of achievement. Alternatively, it may reflect a preponderance of younger researchers, many of whom may be on strong upward trajectories. On the second interpretation, Australia’s proportion of leading psychology researchers is likely to rise in future even if its share of publications does not.

The funding-related findings do not provide a straightforward answer to whether psychology research currently receives the share of support that it merits. The field wins approximately 5% of ARC grants and fellowships, a figure that exceeds the field’s share of leading researchers (but not if researchers in “clinical medicine”, whose funding typically comes from non-ARC sources, are excluded: i.e., 4.89%). It would not be meaningful to compare share of funding with share of publications across fields, given substantial differences in their publication practices. Nevertheless, ARC funding for psychology is substantial, but with noticeably lower rates for industry-partnered Linkage Projects, a finding pointing to ongoing challenges in funding applied psychology research.

The study has several limitations. First, it is constrained by the publication databases it relies on. These under-represent journals published in languages other than English and publication types such as chapters and books, although these are a less prominent publication type in the field and thus unlikely to substantially alter our findings. The databases also do not provide robust ways to assess publication quality, so the present analysis based on journal impact factors is necessarily coarse.

Second, the study delimits “Australian psychology research” according to the listed affiliations of researchers and an identified set of psychology journals. It excludes researchers with Australian backgrounds who have external primary affiliations and the work of Australian-affiliated researchers who publish outside psychology (e.g., in neuroscience, psychiatry or management). The second point implies that our findings under-estimate the absolute (but not necessarily relative) productivity of Australian-affiliated psychology researchers. Conversely, our definition includes non-Australian researchers with Australian affiliations and Australian researchers outside the field of psychology who publish in its journals, which would result in a corresponding over-estimation. Nevertheless, “Australian-affiliated researchers publishing in psychology” draws a meaningful boundary.

Third, the examination of research funding outcomes focuses on research supported by the ARC, omitting large volumes of funding disbursed by NHMRC and MRFF. As these agencies primarily fund research in basic and clinical biomedicine, health science and public health, funding to psychology tends to be relatively modest. The proportion of total Australian competitive research funding flowing to psychology is likely to be significantly over-stated in our analysis, despite being accurate for basic and applied non-medical science.

This study tells an encouraging story about Australian psychological science. The discipline’s rise has been broad and meteoric, it occupies a position of local scientific prominence and global over-achievement, its productivity has a broad base of relatively junior researchers, and it is not self-evidently under-supported by the major national funding agency for basic and applied scientific research. The level of disciplinary success implied by these findings may not be as widely appreciated as it could be.

## Data Availability

All data are available on publicly accessible databases, with the exception of the Web of Science database, which requires a subscription. As a proprietary database, it cannot be made freely available.

## References

[cit0001] Breen, L. J., & Darlaston-Jones, D. (2010). Moving beyond the enduring dominance of positivism in psychological research: Implications for psychology in Australia. *Australian Psychologist*, 45(1), 67–7. 10.1080/00050060903127481

[cit0002] Feather, N. T. (2005). Social psychology in Australia: Past and present. *International Journal of Psychology*, 40(4), 263–276. 10.1080/00207590444000203

[cit0003] Haslam, N., & Baes, N. (2023). Scientific eminence and scientific hierarchy: Bibliometric prediction of fellowship in the Australian Academy of Science. *Scientometrics*, 128(12), 6659–6674. 10.1007/s11192-023-04870-8

[cit0004] Haslam, N., Stratemeyer, M., & Vargas-Saenz, A. (2017). Scholarly productivity and citation impact of academic psychologists in group of eight universities. *Australian Journal of Psychology*, 69(3), 162–166. 10.1111/ajpy.12142

[cit0005] Ioannidis, J. P. A. (2024). August 2024 data-update for “updated science-wide author databases of standardized citation indicators”. *Elsevier Data Repository*, V7. 10.17632/btchxktzyw.7 PMC7567353 33064726

[cit0006] Ioannidis, J. P. A., Baas, J., Klavans, R., & Boyack, K. W. (2019). A standardized citation metrics author database annotated for scientific field. *PLOS Biology*, 17(8), e3000384. 10.1371/journal.pbio.3000384 31404057 PMC6699798

[cit0007] Ioannidis, J. P. A., Boyack, K. W., & Baas, J. (2020). Updated science-wide author databases of standardized citation indicators. *PLOS Biology*, 18(10), e3000918. 10.1371/journal.pbio.3000918 33064726 PMC7567353

[cit0008] Macintyre, S. (2010). *The poor relation: A history of social sciences in Australia*. Melbourne University Press.

[cit0009] Mazzucchelli, T. G., Burton, E., & Roberts, L. (2019). Scholarly productivity and citation impact of Australian academic psychologists. *Australian Journal of Psychology*, 71(3), 305–311. 10.1111/ajpy.12248

[cit0010] Sheehan, P. W. (1996). Anticipations ahead for psychology: Looking from past to future. *Australian Psychologist*, 31(3), 183–190. 10.1080/00050069608260204

[cit0011] Taft, R. (1988). Psychology in Australia. *Annual Review of Psychology*, 39(1), 375–400. 10.1146/annurev.psych.39.1.375

[cit0012] Turtle, A. M. (1985). Psychology in the Australian context. *International Journal of Psychology*, 20(1), 111–128. 10.1002/j.1464-066X.1985.tb00017.x 25825065

